# Prevalence, Antibiogram and Genetic Characterization of *Listeria monocytogenes* from Food Products in Egypt

**DOI:** 10.3390/foods10061381

**Published:** 2021-06-15

**Authors:** Eman E. Abdeen, Walid S. Mousa, Ola. H. Harb, Gehad A. Fath-Elbab, Mohammed Nooruzzaman, Ahmed Gaber, Walaa F. Alsanie, Ahmed Abdeen

**Affiliations:** 1 Department of Bacteriology, Mycology and Immunology, Faculty of Veterinary Medicine, University of Sadat City, Sadat City 32897, Egypt; 2 Department of Animal Medicine and Infectious Diseases, Faculty of Veterinary Medicine, University of Sadat City, Sadat City 32897, Egypt; walid.saad@vet.usc.edu.eg; 3 Faculty of Veterinary Medicine, Veterinarian Teaching Hospital, University of Sadat City, Sadat City 32897, Egypt; ola.harb1373@vet.usc.edu.eg; 4 Animal Health Research Institute (AHRI)—Dokki, Giza 12618, Egypt; gehadfathy172@gmail.com; 5 Department of Pathology, Faculty of Veterinary Science, Bangladesh Agricultural University, Mymensingh 2202, Bangladesh; mohammed.nooruzzaman@bau.edu.bd; 6 Department of Biology, College of Science, Taif University, P.O. Box 11099, Taif 21944, Saudi Arabia; a.gaber@tu.edu.sa; 7 Center of Biomedical Sciences Research (CBSR), Taif University, P.O. Box 11099, Taif 21944, Saudi Arabia; w.alsanie@tu.edu.sa; 8 Department of Clinical Laboratories Sciences, The Faculty of Applied Medical Sciences, Taif University, P.O. Box 11099, Taif 21944, Saudi Arabia; 9 Center of Excellence for Screening of Environmental Contaminants, Benha University, Toukh 13736, Egypt; ahmed.abdeen@fvtm.bu.edu.eg; 10 Department of Forensic Medicine and Toxicology, Faculty of Veterinary Medicine, Benha University, Toukh 13736, Egypt

**Keywords:** *L. monocytogenes*, virulence genes, foods, sequencing, antibiogram

## Abstract

World Health Organization classified *Listeria monocytogenes* as a major notable foodborne pathogen associated with high mortality and hospitalization. The study reports the prevalence, antibiogram, virulence determination and genetic characterization of *L. monocytogenes* from different food products. A total of 250 food samples, fifty samples each from raw milk, ice cream, minced meat, fish fillet and sausage were collected from the Menoufiya governorate in Egypt. *L. monocytogenes* was detected in 17 (6.8%) of the tested food samples including minced meat (14%), fish fillet (8%), sausage (6%) and raw milk (6%). The antimicrobial susceptibility assay of 17 *L. monocytogenes* isolates against seventeen antibiotics belonging to eight antibiotics classes revealed a high susceptibility to norfloxacin (82.3%), amoxicillin-clavulanic acid (76.4%), cefotaxime (70.5%), erythromycin (64.6%), amoxicillin (64.6%), gentamicin (58.7%) and vancomycin (58.7%). While, high resistance was observed against oxytetracycline (76.4%), trimethoprim-sulfamethoxazole (76.4%), chloramphenicol (70.5%), doxycycline (64.6%), levofloxacin (41.2%) and azithromycin (41.2%). Of note, all *L. monocytogenes* isolates were multidrug-resistant. The multiplex PCR successfully amplified *L. monocytogenes* in all tested isolates. Screening of the five virulence-related genes revealed the *hlyA* and *iap* as the most prevalent genes followed by *actA* gene, however, the *inlA* and *prfA* genes were not detected in any of the studied isolates. The partial *16S rRNA* gene sequencing of three *L. monocytogenes* isolates showed a high nucleotide similarity (99.1–99.8%) between the study isolates and various global clones, and phylogenetic analysis clustered these *L. monocytogenes* strains with other Listeria species including *L. welshimeri*, *L. seeligeri* and *L. innocua*. This study demonstrates the impact of *L. monocytogenes* as a major contaminant of various food products and suggests more attention to the awareness and hygienic measures in the food industry.

## 1. Introduction

*Listeria monocytogenes* is a ubiquitous foodborne pathogen associated with high hospitalization and outbreaks of food-borne illness worldwide [1]. *L. monocytogenes* causes listeriosis in humans and animals and can be found in varieties of foods and dairy products [2]. The bacteria is also considered as a potential contaminant source for chilled and marine food products [3] and unpasteurized dairy products and has been detected in recent outbreaks and sporadic cases of listeriosis [4]. In a dairy herd, contaminated teat cups often serve as a potential source in the transmission of *L. monocytogenes* [2]. In line with this, three *L. monocytogenes* variant clones, the hypervirulent clones Clonal Complexes 1 (CC1) and hypovirulent clones (CC9 and CC121), were reported in humans that were closely associated with the dairy and meat products, respectively [5].

The pathogenicity of *L. monocytogenes* is largely determined by a group of virulence genes. *L. monocytogenes* strains from dairy herds carried a number of virulence markers including the Listeria pathogenicity islands (LIPI) 3 and LIPI-4 that were associated with severe human infections [4]. Virulence factors are associated with crucial stages of adhesion, invasion, reproduction, motility and intercellular spread into host cells and therefore play a key role in virulence and resistance against host immunity [6]. The major determinant virulence genes include internalins encoded by *inlA*, *inlC* and *inlJ* genes, listeriolysin encoded by *hlyA* gene, actin encoded by *actA* gene and the invasion associated protein encoded by *iap* gene [7]. The *hlyA*, *plcB* and *prfA* virulence genes have been found in *L. monocytogenes* strains recovered from blood and milk isolates [2]. Furthermore, the *prfA* virulence gene is a transcriptional activator identified among pathogenic *L. monocytogenes* [8]. Moreover, biofilm activity is commonly associated with *L. monocytogenes* of milk origin that carry a variety of virulence and antibiotics resistance genes [2]. Traditionally, *Listeria* diagnosis is primarily based on isolation and biochemical characterization, which is time consuming. Molecular approaches such as PCR and multiplex PCR (mPCR) provide rapid and specific techniques for the diagnosis of *L. monocytogenes* targeting specific genes [9].

Antimicrobial resistance is an important public health issue and one of the highest priorities of the World Health Organization (WHO). The growing level of antimicrobial resistance has led to higher patient morbidity and mortality rates and increased healthcare expenditure over the last decades [10,11]. The multidrug-resistant *L. monocytogenes* particularly in ready-to-eat foods is being considered as a public health indicator especially among the high-risk groups. It is highly recommended to build awareness about the importance of food safety regulations as well as drugs used in humans and animals [12]. The nucleotide sequence analysis of *L. monocytogenes* is an effective modern tool for genotyping and analysis of the relatedness of *Listeria* species with other local or global lineages. A surveillance study conducted in the United States reported a substantial genetic similarity between *L. monocytogenes* strains from milk tanks with virulent strains isolated from dairy products, which were associated with the outbreaks of food-borne illness in humans [4].

The Menoufiya governorate of Egypt is a densely populated rural governorate where people consume many famous and popular ready-to-eat foods such as minced meat meal, fish dishes, milk products and ice cream. Such raw uncooked or undercooked foods can be a potential source of pathogenic *L. monocytogenes* in humans. This study describes the virulence and antibiotic resistance profiles of *L. monocytogenes* detected in various ready-to-eat food products from Egypt. In addition, partial nucleotide sequence analysis of the *16S rRNA* gene of three *L. monocytogenes* isolates was performed to correlate the genetic similarities between the study isolates and various global clones having potential public health implications.

## 2. Materials and Methods

### 2.1. Ethics Statement

This study followed the guidelines of the Ethics Committee and current legislation on research and ethical approval of the Faculty of Veterinary Medicine (Local ethical approval), University of Sadat City, Egypt.

### 2.2. Study Area

This study was carried out in the Monufiya governorate in Lower Egypt. The majority of residents in this governorate live in rural areas, with an urbanization rate of only 20.6% [13]. The area has a high population density and people consume a lot of ready-to-eat popular foods such as minced meat, fish dishes, raw milk products and ice cream.

### 2.3. Samples Collection and Processing

A total of 250 food products (50 samples each from raw milk, ice cream, minced meat from beef, fish fillet and sausage) were collected from different local markets at Menoufiya governorate in Egypt from January to August 2020. Each sample was collected separately in a sterile plastic bag and transferred to the laboratory in cooled condition.

### 2.4. Phenotypic Isolation and Identification of Listeria from Food Products

Twenty-five grams of food sample was homogenized with 9 mL of nutrient broth using a blender. The harvested homogenate was firstly pre-enriched in 225 mL of Buffered Listeria Enrichment Broth with pyruvate and incubated at 30 °C for 48 h, then cultured in specific Oxford medium, CHROM agar and sheep blood agar (Himedia, India) for 48 h at 35 °C as described by FDA BAM and ISO 11290 method [14]. Characteristic colonies of *L. monocytogenes* were identified on different agar media. Morphological and biochemical characteristics of the bacteria were analyzed using Gram staining, catalase test, sugar fermentation test and motility test according to FDA BAM and ISO 11290 method [14]. The CAMP (Christie–Atkins–Munch-Peterson) test was performed as described previously [15] using standard hemolytic *Staphylococcus aureus* strain (MT211620), which was streaked on blood agar in a straight line across the center of the plate, then the *L. monocytogenes* strain was streaked in a direction perpendicular or vertical to *S. aureus* without touching the *S. aureus* culture. Then the plates were incubated at 37 °C for 18–24 h and checked for β-hemolysis which appeared as an arrowhead, circle or rectangle shape in CAMP-positive species.

### 2.5. Antibiogram Profile of L. monocytogenes Isolates Recovered from Food Products

The Kirby-Bauer disk diffusion method was used to analyze the antibiogram profile of *L. monocytogenes* isolates. The bacterial suspension was adjusted to a 0.5 McFarland. Seventeen different antibiotic disks (Oxoid Ltd., Basingstoke, UK) belonging to eight different antibiotic groups were used. The antibiotics included amikacin (AK) 30 µg, ciprofloxacin (CIP) 5 µg, nalidixic acid (NA) 30 µg, chloramphenicol (C) 30 µg, doxycycline (DO) 30 µg, cefotaxime (CTX) 30 µg, trimethoprim-sulfamethoxazole (SXT) 12.5/23.75 µg, amoxicillin/nalidixic acid (AMC) 20/10 µg, levofloxacin (LEV) 5 µg, norfloxacin (NOR) 10 µg, azithromycin (AZM) 15 µg, danofloxacin (DA) 2 µg, oxytetracycline (T) 30 µg, erythromycin (E) 15 µg, gentamicin (CN) 10 µg, vancomycin (VA) 30 µg and amoxicillin (AX) 30 µg. The result was interpreted as resistant, intermediate or susceptible based on the inhibitory zone as described by the Clinical and Laboratory Standards Institute (CLSI) [16]. The strains displaying resistance to at least three antibiotic classes were considered multidrug-resistant (MDR) [17]. The *L. monocytogenes* strain LMEGY1 was used as a quality control organism in antimicrobial susceptibility determination. All samples were tested twice.

### 2.6. Molecular Detection of L. monocytogenes from Food Products

DNA was extracted using QIAamp DNA Mini Kit (Qiagen, Hilden, Germany). The list of primers used in the study is provided in Table 1. Taq PCR Master Mix Kit (Qiagen, Hilden, Germany) was used in the PCR. A 50 µL reaction was prepared to contain 25 µL PCR Master Mix, 1 µL (10 pmol/µL) of each primer, 2 µL (50 ng/µL) DNA and the remaining volume needed to reach 50 µL was adjusted with deionized water. The following thermal profile was used: initial denaturation at 95 °C for 3 min; 35 cycles each consisting of denaturation at 94 °C for 30 s, annealing at 53 °C for 15 s and extension at 72 °C for 90 s; and final extension at 72 °C for 7 min. PCR products (15 µL) were analyzed by agarose gel (1.5%) electrophoresis and visualized under UV light in a gel documentation system. The *L. monocytogenes* LMEGY1 strain was used as a positive control in the PCR.

### 2.7. Sequencing and Phylogenetic Analysis of L. monocytogenes from Food Products

Three PCR amplicon of the *16S rRNA* gene of *L. monocytogenes*, each from raw milk, fish fillet and minced meat were purified using Gene JET PCR Purification kit (Thermo Scientific, Waltham, MA, USA). The purified PCR products were sequenced from a commercial laboratory (GATC Biotech Company, Konstanz, Germany) in both directions. Nucleotide and amino acid sequence homology analysis among studied isolates and global strains was performed using BLAST 2.0 and PSI-BLAST search programs (http://www.ncbi.nlm.nih.gov/, accessed on 20 March 2021), respectively. Multiple alignments with reference strains as well as the deduction of amino acid sequences were performed using the BioEdit [19], CLUSTALX [20], ClustalW [21], ClustalV [22] and MegAlign software (DNASTAR, Lasergene^®^, Version 7.1.0, Madison, WI, USA) [23]. A neighbor-joining phylogenetic tree was built using MegAlign software. A random bootstrapping value of 111 was applied [21]. The partial nucleotide sequences of the three *L. monocytogenes* strains from the fish fillet, minced meat and raw milk were submitted in the GenBank with accession number MW090062, MW090063 and MW090064, respectively.

### 2.8. Statistical Analysis

To show the multidrug resistance profile of *L. monocytogenes* isolates, an UpSetR plot was prepared using an online platform (https://gehlenborglab.shinyapps.io/upsetr/, accessed on 21 April 2021).

## 3. Results

### 3.1. Prevalence of Listeria Species and L. monocytogenes in Different Food Products

The overall prevalence of *L. monocytogenes* was 6.8% (*n* = 17), while other *Listeria* species were as followed: *L. innoca* 3.2% (*n* = 8), *L. grayi* 2.4% (*n* = 6), *L. ivanovii* 0.4% (*n* = 1) and *L. welshimeri* 0.8% (*n* = 2). Among the different food samples, *L. monocytogenes* was most commonly detected in minced meat (14%) followed by fish fillet (8%), sausage (6%) and milk (6%) (Table 2). Analysis of the ice cream samples yielded no *L. monocytogenes*.

Typical colony characters of *L. monocytogenes* on Oxford medium appeared as gray to black color colonies bounded by a black halo. A green to bluish colony was observed in CHROM agar medium. On the 5% sheep blood agar medium, *L. monocytogenes* showed a clear narrow β-hemolysis. A series of biochemical tests were conducted to confirm and differentiate *L. monocytogenes* and other *Listeria* species (Supplementary Table S1). *L. monocytogenes* appeared as Gram positive coccobacilli, catalase and CAMP test positive as well as fermented rhamnose with acid production, while negative for fermentation of mannitol and xylose (Supplementary Table S1).

### 3.2. Antibiogram of L. monocytogenes Isolates from Food Products

Antimicrobial resistance profiles of *L. monocytogenes* isolates were tested against seventeen antibiotics belonging to eight different antibiotic classes using the disc diffusion method (Table 3). *L. monocytogenes* isolates showed high susceptibility to β-lactams (amoxicillin-clavulanic acid, cefotaxime and amoxicillin), norfloxacin, erythromycin, gentamicin and vancomycin. While, high resistance was observed against tetracycline (oxytetracycline and doxycycline), trimethoprim-sulfamethoxazole and chloramphenicol (Table 3). An overall similar antimicrobial resistance profile was observed in *L. monocytogenes* isolates from different sources such as milk, fish fillet, sausage and minced meat (Supplementary Table S2).

**Figure 1:**
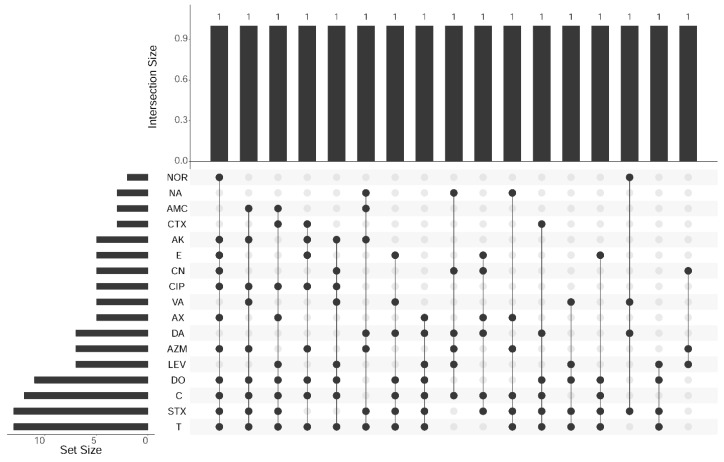
The multidrug-resistant profiling of *L. monocytogenes* isolates from food products. Note: amikacin (AK), ciprofloxacin (CIP), nalidixic acid (NA), chloramphenicol (C), doxycycline (DO), cefotaxime (CTX), trimethoprim-sulfamethoxazole (SXT), amoxicillin/nalidixic acid (AMC), levofloxacin (LEV), norfloxacin (NOR), azithromycin (AZM), danofloxacin (DA), oxytetracycline (T), erythromycin (E), gentamicin (CN), vancomycin (VA) and amoxicillin (AX).

Next, multidrug resistance profiles of the 17 *L. monocytogenes* isolates were tested. All *L. monocytogenes* isolates were multidrug-resistant against 3 to 11 different antibiotics belonging to 3 to 7 antibiotic classes in 17 different combinations (Figure 1).

### 3.3. Screening of Different Virulence Genes in L. monocytogenes from Food Products

**Figure 2:**
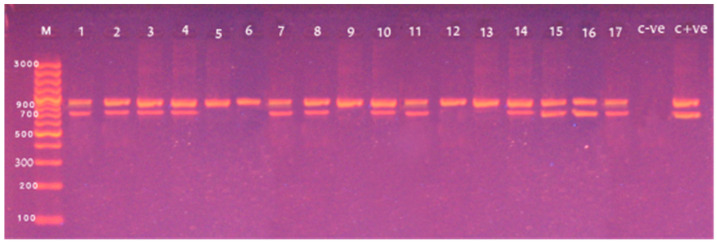
Amplification of *16S rRNA* and *hlyA* genes of *L. monocytogenes* at 938 bp and 702 bp, respectively, by multiplex PCR. Lane M: 100 bp DNA ladder; Lane 1–17 positive samples for *16S rRNA*; Lane 1–3 (raw milk), 4, 7–8 (fish fillet), 10–11 (sausage) and 14–17 (minced meat) were positive for the *hlyA* gene; Lane C −ve: Control Negative; Lane C +ve: Control Positive.

**Figure 3:**
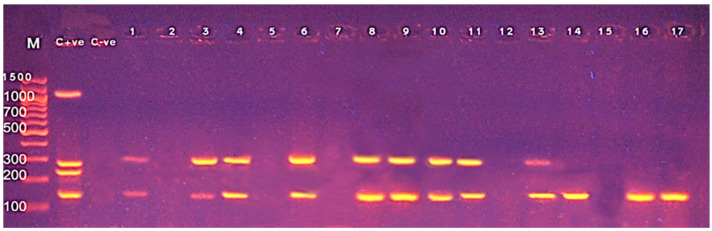
Amplification of *inlA*, *actA*, *prfA* and *iap* genes of *L. monocytogenes* at 255 bp, 268 bp, 1060 bp and 131 bp, respectively, by multiplex PCR. Lane M: 100 bp DNA ladder; Lane C +ve: Control Positive; Lane C -ve: Control Negative; Lane 1, 3, 4, 6 (minced meat), 8–11 (fish fillet), 13–14 (raw milk), 16–17 (sausage) were positive for the *iap* gene at 131 bp. While lane 1, 3, 4 (minced meat), 6, 8 (raw milk), 9, 10 (fish fillet) and 11, 13 (sausage) were positive for the *actA* gene at 268 bp. The *prfA* and *inlA* genes were not detected in any samples.

Next, we applied multiplex PCR for molecular detection of the *L. monocytogenes* and screening five virulence-associated genes of the bacteria. Amplification of 938 bp fragment of the *16S rRNA* gene was found in all of the 17 tested isolates, confirming the *L. monocytogenes* species (Figure 2). While, the screening of five virulence-associated genes in *L. monocytogenes* revealed the *hlyA* (*n* = 12, 70.6%) and *iap* (*n* = 12, 70.6%) as the most prevalent genes followed by the *actA* gene (*n* = 9, 52.9%) (Figure 2 and Figure 3). However, *inlA* and *prfA* genes were absent in all of the tested isolates. Of note, the simultaneous presence of three virulence genes (*hlyA*, *iap* and *actA*) in six isolates, two of the three virulence genes (*hlyA*, *iap* or *actA*) in six isolates was also detected. Taken together, *L. monocytogenes* isolates from food products carried multiple virulence genes, indicating their pathogenic potential.

### 3.4. Sequence Analysis of L. monocytogenes Isolates from Food Products

**Figure 4:**
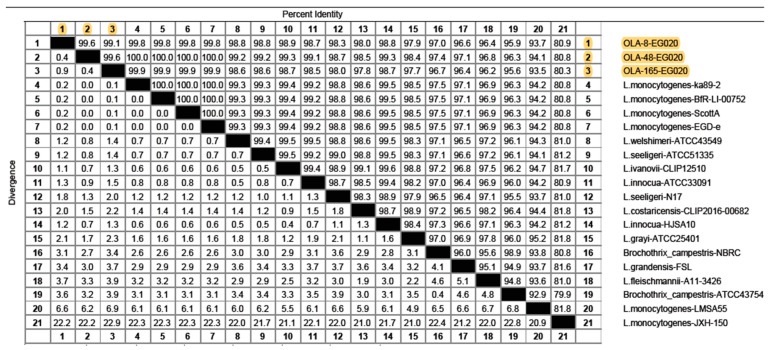
Nucleotide percent identity and divergence between the *L. monocytogenes* from raw milk, fish fillet and minced meat and related strains retrieved from GenBank. Sequences (highlighted in yellow color) from raw milk (OLA-165-EG020, accession number MW090064), fish fillet (OLA-8-EG020, accession number MW090062) and minced meat (OLA-48-EG020, accession number MW090063) were generated in this study.

The *16S rRNA* sequence analysis was used for evaluating the genetic similarity of *L. monocytogenes* isolates from fish fillet, minced meat and raw milk samples with global strains. The partial sequence data were submitted to the GenBank with accession number MW090062, MW090063 and MW090064. The study isolates showed a very high nucleotide similarity (99.1–99.8%) with *L. monocytogenes* strains isolated in Turkey and Germany from minced meat and food origin with accession number MT633107.1 (strain: ka89-2), and CP054846 (strain: BfR-LI-00752), respectively (Figure 4). Additionally, the near identity was also noticed with *L. monocytogenes* strain identified from the Massachusetts listeriosis outbreak in the USA (accession number CP023862, strain: ScottA). As well as one strain from rabbit tissue from United Kingdom (accession number CP023861, strain: EGD-e) had identical nucleotides similarity (99.6–100%) with our strains from food samples.

**Figure 5:**
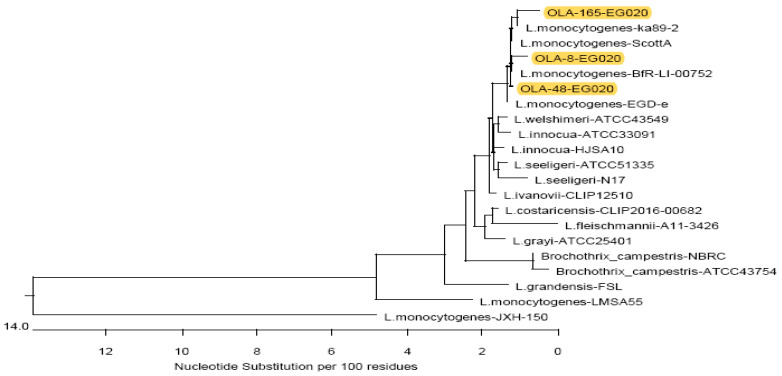
Neighbor-joining phylogenetic tree of *Listeria monocytogenes* from raw milk, fish fillet and minced meat based on the partial *16S rRNA* gene sequences. Sequences (highlighted in yellow color) from raw milk (OLA-165-EG020, accession number MW090064), fish fillet (OLA-8-EG020, accession number MW090062) and minced meat (OLA-48-EG020, accession number MW090063) were generated in this study.

The phylogenetic tree clustered our three *L. monocytogenes* strains with other *Listeria* species including *L. welshimeri* (strain ATCC 43549), *L. seeligeri* (strain ATCC 51335) and *L. innocua* (ATCC 33091) strains from China with accession number JF967629.1, JF967627.1 and JF967626.1, respectively (Figure 5). Furthermore, several *Listeria* strains shared sequence homology with the study isolates including one isolate from braised chickens in China (MT781377.1, strain HJSA10), four strains from different countries and localities including *L. ivanovii* from the USA (NR_036808.1, strain CLIP 12510), *L. seeligeri* from Turkey (MK490993.1, strain N17), *L. costaricensis* from France (MK174378.1, strain CLIP 2016/00682) and *L. grayi* from Switzerland (JN852815.1, strain ATCC 25401). On the other hand, a distinct diversity was detected in the phylogenic tree with two *L. monocytogenes* isolates from salads in Nigeria (KY053294.1, strain LMSA55) and deep-sea sediment in the Pacific Ocean (KR012147.1, strain JXH-150) (Figure 4 and Figure 5).

## 4. Discussion

*Listeria monocytogenes* possesses a significant public health significance due to frequent contamination of food products [24]. In Egypt, *L. monocytogenes* enters into human food chain primarily through contaminated meat and chicken products [9]. In particular, the ability of *L. monocytogenes* to grow in the refrigerator or cooled condition in varieties of food products makes the pathogen difficult to control [3]. Detection of the bacteria in food products at retail outlets indicates a major defect in the quality control measures [25].

Here, the overall prevalence rate of *L. monocytogenes* from 250 different food products was 6.8%, while other *Listeria* species, *L. innoca*, *L. grayi*, *L. ivanovii* and *L. welshimeri* were detected in 3.2%, 2.4%, 0.4% and 0.8% of the samples, respectively. More specifically, the *L. monocytogenes* was found in 14%, 8%, 6% and 6% of minced meat, fish fillet, sausage and raw milk samples, respectively, while none of the ice cream samples showed positive growth of the bacteria. Our study corroborates a recent study that demonstrated 6% and 4% prevalences of *L. monocytogenes* in urban and rural areas of Egypt, respectively [25]. The study also detected a high prevalence of *L. monocytogenes* in minced meat (56%), poultry meat (18%), tilapia fish (6%) and raw milk (10%) in Egypt [25]. A similar study in the Czech Republic during 2004–2008 reported *L. monocytogenes* in 5.2%, 3.4% and 1.8% of the delicatessen, meat and dairy products, respectively [26]. In contrast, Oliveira and colleagues isolated *L. monocytogenes* in 17.9% of meat cuts and 8.3% from cuts after packing meat samples [27]. In Ethiopia, the prevalence of *Listeria* species and *L. monocytogenes* in ready-to-eat foods was found 25% and 6.25%, respectively [12]. A similar study in Portugal detected a 7% prevalence of *L. monocytogenes* in 1035 food products (milk, meat, fish, flour) indicating a potential risk for consumers [28]. The variation in the detection rate of *L. monocytogenes* among different studies in varieties of food products could be explained by types of foods, sample size, geographic area and the degree of sanitary measures applied during food processing and manufacture.

The antibiogram profile of the 17 *L. monocytogenes* isolates against seventeen antibiotics displayed a high sensitivity to norfloxacin (82.3%), amoxicillin-clavulanic acid (76.4%), cefotaxime (70.5%), erythromycin (64.6%), amoxicillin (64.6%), gentamicin (58.7%) and vancomycin (58.7%). While, high resistance was perceived against oxytetracycline (76.4%) and trimethoprim-sulfamethoxazole (76.4%), chloramphenicol (70.5%), doxycycline (64.6%), levofloxacin (41.2%) and azithromycin (41.2%). The antimicrobial profiles of *L. monocytogenes* strains recovered from four different sources such as raw milk, fish fillet, sausage and minced meat were broadly similar. Moreover, all of the 17 *L. monocytogenes* isolates were multidrug-resistant. Our findings corroborate an earlier study [29] which showed high resistance of *L. monocytogenes* isolates against penicillin, amoxicillin/clavulanic acid, tetracycline and chloramphenicol. Moreover, *L. monocytogenes* isolated from fish and fish products in India showed resistance to multiple antibiotics [30]. High susceptibility of *L. monocytogenes* to several antibiotics including amoxicillin, cephalothin, cloxacillin and sulfamethoxazole and high resistance rate against penicillin, nalidixic acid, tetracycline and chloramphenicol were also found in isolates from several ready-to-eat food products of milk, meat and fish origin [12]. Another study also reported antimicrobial resistance to two or more antibiotics of 36 *L. monocytogenes* from raw milk which indicates a public health threat to the consumers [31]. On the contrary, a recent study showed 100% susceptibility of the *L. monocytogenes* isolates to most of the tested antibiotics which emphasized the need for the continuous monitoring of antimicrobial susceptibility pattern and their effects on public health [27]. Another study found that resistance to penicillin and erythromycin were common in 44.4% and 60% of the *L. monocytogenes* isolated from milk and clinical specimens, respectively [2].

The PCR offers rapid and sensitive detection of *L. monocytogenes* in food products which is crucial in the food industry [32]. We employed a multiplex PCR technique to detect *L. monocytogenes* by amplifying the *16S rRNA* gene and screened five virulence genes of *L. monocytogenes*. Amplification of the *16S rRNA* genes was found in all of the tested isolates. Screening of the five virulence genes showed that the *hlyA*, *iap* and *actA* were the most detected virulence genes with the prevalence rate of 70.6%, 70.6% and 52.9%, respectively. Of note, simultaneous detection of the three virulence genes (*hlyA*, *iap* and *actA*) was found in six *L. monocytogenes* isolates. Our findings support a recent study in Egypt that detected four virulence genes (*inlA*, *actA*, *prfA* and *hlyA*) in *L. monocytogenes* isolates from animal food products [25]. Harb and colleagues showed that the mPCR targeting the *16S rRNA* and *hlyA* genes can effectively detect *L. monocytogenes* in food samples [33]. In a comparative study in Nigeria, Usman and colleagues used the mPCR to detect multiple virulence-related genes (*prf A*, *inlA*, *hlyA*, *actA* and *iap*) in *L. monocytogenes* from milk and milk products where 25% of them carried one or two of the virulence genes [18]. In Egypt, Abdellrazeq and colleagues examined five virulence genes (*prfA*, *hlyA*, *actA*, *inlA* and *prs*) in *L. monocytogenes* from various fish types by mPCR and detected *prs* gene in all tested isolates while only seven isolates carried other virulence genes [34]. In India, Kaur and colleagues tested 335 food samples (chicken, pork and fish) from various retail outlets and found that all *L. monocytogenes* isolates possess the *prfA*, *plcA*, *actA*, *hlyA* and *iap* virulence-related genes [35]. Furthermore, Haj Hosseini and colleagues demonstrated the *prfA* gene in all *L. monocytogenes* from contaminated foods in Iran [8]. Several virulent genes were also detected in *L. monocytogenes* from fish and fish products in India [30]. Of note, the two virulence-related genes, *prfA* and *inlA* were not found in any of the tested isolates. Our observations might be attributed to complete absence or presence of sequence variations in sites targeted by applied primers within genes found in *L. monocytogenes* isolates of our study. Since we detected no hemolytic activity differences on sheep blood agar between our all isolates and the reference strain used *L. monocytogenes* strain LMEGY1 with an intact *prfA* gene, the lack of *prfA* gene among our strains can however be ruled out.

The partial sequencing of the *16S rRNA* gene was performed to assess the genetic homology among *L. monocytogenes* isolates from milk, fish fillet and minced meat as well as among related global sequences. Our findings showed a high nucleotide similarity (99.1–99.8%) between *L. monocytogenes* strains from various global clones. The phylogenetic tree clustered the three *L. monocytogenes* isolates from this study with other *Listeria* species including *L. welshimeri*, *L. seeligeri* and *L. innocua* strains. In addition, an apparent diversity was found in the phylogenetic tree with some other strains. These findings corroborate with other studies [36,37] which recorded a close association (more than 99% nucleotide similarity) between the members of *Listeria* species and this highlighted the significance of the *16S rRNA* gene in differentiating *Listeria* species. Nucleotide sequencing and multilocus sequence typing (MLST) techniques are suggested to be more precise techniques for recognizing the clonal complexes (CC) of *L. monocytogenes* strains and phylogenetic characters among different *Listeria* strains in a population [38]. The sequencing analysis of the *16S rRNA* gene of *L. monocytogenes* recovered from bulk tank milk in the USA described a high genetic diversity with many strains encoding virulence markers that were linked with serious human infections [4]. The comparison with global clones grouped the *L. monocytogenes* isolates into two distinct clusters, linage A, which are typically associated with epidemic listeriosis, and lineage B, which are mostly associated with sporadic cases of listeriosis [18]. Thus, our study emphasizes that these local isolates may have a potentials public health concern for humans through the food chain.

## 5. Conclusions

The study demonstrates the impact of *L. monocytogenes* as a major contaminant of various food products and the need for more attention about the awareness and hygienic measures in the food industry. Most of the *L. monocytogenes* from food products were multidrug-resistant, adding further burden to the existing global antimicrobial resistance problems. The sequencing analysis reported a high nucleotide sequence similarity of the study isolates with many global clones, indicating the widespread circulation of such strains between different countries via the food trading industry. Therefore, evidence-based recommendations and continuous education for workers particularly in the food industry are necessary to prevent food contamination and the emergence of resistant strains.

## Figures and Tables

**Table 1:** List of PCR primers used in the molecular detection and characterization of *L. monocytogenes*.

Targets	Primers	Sequences (5′ to 3′)	Amplicon Size (bp)	Reference
*16S rRNA*	Forward	CTC CAT AAA GGT GAC CCT	938	[18]
	Reverse	CAG CMG CCG CGG TAA TWC		
*hlyA*	Forward	CCT AAG ACG CCA ATC GAA	702	
	Reverse	AAG CGCTTG CAA CTG CTC		
*inlA*	Forward	AGA TCT AGA CCA AGT TAC AAC GCT TCA	255	
	Reverse	TAA TAT CAT TTG CTG TTT TAT CTG TC		
*actA*	Forward	ACG TGA AGT AAG TCACGT GAT ATT G	268	
	Reverse	ACG TGA AGT AAG CTC ACG TGA TAT TG		
*prfA*	Forward	ACC GCT CAG AAA AGT TCT TC	1060	
	Reverse	TCT TGT TCT ATT ATGTCT AGC		
*iap*	Forward	ACA AGC TGC ACC TGT TGC AG	131	
	Reverse	TGA CAG CGT TGT TAG TAG CA		

**Table 2:** Prevalence of *Listeria monocytogenes* in food products.

Raw Milk (*n* = 50)		Ice Cream (*n* = 50)		Minced Meat (*n* = 50)		Fish Fillet (*n* = 50)		Sausage (*n* = 50)	
No.	%	No.	%	No.	%	No.	%	No.	%
3	6	0	0	7	14	4	8	3	6

% was estimated according to the total number of each sample (50).

**Table 3:** Antimicrobial resistance profiles of *L. monocytogenes* isolates from food products.

Antibiotics	Antimicrobial Classes	Resistant		Intermediate		Sensitive	
		No	%	No	%	No	%
Amoxicillin-Clavulanic acid (AMC) 20/10 µg	β-lactams	3	17.7	1	5.9	13	76.4
Cefotaxime (CTX) 30 µg	β-lactams	3	17.7	2	11.8	12	70.5
Amoxicillin (AX) 30 µg	β-lactams	5	29.5	1	5.9	11	64.6
Norfloxacin (NOR) 10 µg	Fluoroquinolones	2	11.8	1	5.9	14	82.3
Ciprofloxacin (CIP) 5 µg	Fluoroquinolones	5	29.4	2	11.8	10	58.8
Levofloxacin (LEV) 5 µg	Fluoroquinolones	7	41.2	2	11.8	8	47
Danofloxacin (DA) 2 µg	Fluoroquinolones	7	41.2	2	11.8	8	47
Nalidixic acid (NA) 30 µg	Fluoroquinolones	3	17.7	10	58.8	4	23.5
Amikacin (AK) 30 µg	Aminoglycosides	5	29.4	1	5.9	11	64.6
Gentamicin (CN) 10 µg	Aminoglycosides	5	29.5	2	11.8	10	58.7
Erythromycin (E) 15 µg	Macrolides	5	29.5	1	5.9	11	64.6
Azithromycin (AZM) 15 µg	Macrolides	7	41.2	7	41.2	3	17.7
Doxycycline (DO) 30 µg	Tetracycline	11	64.6	3	17.7	3	17.7
Oxytetracycline (T) 30 µg	Tetracycline	13	76.4	1	5.9	3	17.7
Chloramphenicol (C) 30 µg	Chloramphenicol	12	70.5	2	11.8	3	17.7
Trimethoprim-Sulfamethoxazole (SXT) 12.5/23.75 µg	Sulfonamides	13	76.4	2	11.8	2	11.8
Vancomycin (VA) 30 µg	Glycopeptides	5	29.5	2	11.8	10	58.7

% was estimated according to the total number of *L. monocytogenes* isolates (*n* = 17).

## Data Availability

All authors agree that the data presented in this study are openly available through MDPI publisher platform or others without any restriction.

## Supplementary Materials

The following are available online at https://www.mdpi.com/article/10.3390/foods10061381/s1, Table S1: Biochemical identification of *Listeria monocytogenes* and other *Listeria* species, Table S2: Antimicrobial resistance profiles of *Listeria monocytogenes* isolates from food products.

## References

[1] Osman K.M., Kappell A.D., Fox E.M., Orabi A., Samir A. (2020). Prevalence, pathogenicity, virulence, antibiotic resistance, and phylogenetic analysis of biofilm producing *Listeria monocytogenes* isolated from different ecological niches in Egypt: Food, humans, animals, and environment. Pathogens.

[2] Skowron K., Wałecka-Zacharksa E., Grudlewska K., Wiktorczyk N., Kaczmarek A., Gryń G., Kwiecińska-Piróg J., Juszczuk K., Paluszak Z., Kosek-Paszkowska K. (2019). Characteristics of *Listeria monocytogenes* strains isolated from milk and humans and the possibility of milk-borne strains transmission. Polish J. Microbiol..

[3] Vinothkumar R., Arunagiri K., Sivakumar T. (2013). Studies on pathogenic *Listeria monocytogenes* from marine food resources. Int. J. Curr. Microbiol. Appl. Sci..

[4] Kim S.W., Haendiges J., Keller E.N., Myers R., Kim A., Lombard J.E., Karns J.S., Van Kessel J.A.S., Haley B.J. (2018). Genetic diversity and virulence profiles of *Listeria monocytogenes* recovered from bulk tank milk, milk filters, and milking equipment from dairies in the United States (2002 to 2014). PLoS ONE.

[5] Maury M.M., Bracq-Dieye H., Huang L., Vales G., Lavina M., Thouvenot P., Disson O., Leclercq A., Brisse S., Lecuit M. (2019). Hypervirulent *Listeria monocytogenes* clones’ adaption to mammalian gut accounts for their association with dairy products. Nat. Commun..

[6] Camejo A., Carvalho F., Reis O., Leitão E., Sousa S., Cabanes D. (2011). The arsenal of virulence factors deployed by *Listeria monocytogenes* to promote its cell infection cycle. Virulence.

[7] Ward T.J., Gorski L., Borucki M.K., Mandrell R.E., Hutchins J., Pupedis K. (2004). Intraspecific phylogeny and lineage group identification based on the *prfA* virulence gene cluster of *Listeria monocytogenes*. J. Bacteriol..

[8] Haj Hosseini A., Sharifan A., Tabatabaee A. (2014). Isolation of *Listeria monocytogenes* from meat and dairy products. J. Med. Microbiol. Infect. Dis..

[9] El-Malek A.M.A., Ali S.F.H., Hassanein R., Mohamed M.A., Elsayh K.I. (2010). Occurrence of *Listeria* species in meat, chicken products and human stools in Assiut city, Egypt with PCR use for rapid identification of *Listeria monocytogenes*. Vet. World.

[10] Bloom G., Merrett G.B., Wilkinson A., Lin V., Paulin S. (2017). Antimicrobial resistance and universal health coverage. BMJ Glob. Health.

[11] Friedman N.D., Temkin E., Carmeli Y. (2016). The negative impact of antibiotic resistance. Clin. Microbiol. Infect..

[12] Garedew L., Taddese A., Biru T., Nigatu S., Kebede E., Ejo M., Fikru A., Birhanu T. (2015). Prevalence and antimicrobial susceptibility profile of *Listeria* species from ready-to-eat foods of animal origin in Gondar Town, Ethiopia. BMC Microbiol..

[13] Monufia Governorate—Wikipedia. https://en.wikipedia.org/wiki/Monufia_Governorate.

[14] Scotter S.L., Langton S., Lombard B., Schulten S., Nagelkerke N., In’t Veld P.H., Rollier P., Lahellec C. (2001). Validation of ISO method 11290. Part 1—Detection of *Listeria monocytogenes* in foods. Int. J. Food Microbiol..

[15] Wilkinson H.W. (1977). CAMP-disk test for presumptive identification of group B streptococci. J. Clin. Microbiol..

[16] CLSI (2017). Performance Standards for Antimicrobial Susceptibility Testing.

[17] Magiorakos A.P., Srinivasan A., Carey R.B., Carmeli Y., Falagas M.E., Giske C.G., Harbarth S., Hindler J.F., Kahlmeter G., Olsson-Liljequist B. (2012). Multidrug-resistant, extensively drug-resistant and pandrug-resistant bacteria: An international expert proposal for interim standard definitions for acquired resistance. Clin. Microbiol. Infect..

[18] Usman U.B., Kwaga J.K.P., Kabir J., Olonitola O.S., Radu S., Bande F. (2016). Molecular Characterization and Phylogenetic Analysis of *Listeria monocytogenes* isolated from milk and milk products in Kaduna, Nigeria. Can. J. Infect. Dis. Med. Microbiol..

[19] Hall T., Biosciences I., Carlsbad C. (2011). BioEdit: An important software for molecular biology. GERF Bull. Biosci..

[20] Ball N.L., Adams C.R., Xia W. Overcoming the elusive problem of IS/IT alignment: Conceptual and methodological considerations. Proceedings of the Ninth Americas Conference on Information Systems.

[21] Thompson J.D., Higgins D.G., Gibson T.J. (1994). CLUSTAL W: Improving the sensitivity of progressive multiple sequence alignment through sequence weighting, position-specific gap penalties and weight matrix choice. Nucleic Acids Res..

[22] Higgins D.G., Sharp P.M. (1989). Fast and sensitive multiple sequence alignments on a microcomputer. Bioinformatics.

[23] Kumar S., Tamura K., Nei M. (2004). MEGA3: Integrated software for molecular evolutionary genetics analysis and sequence alignment. Brief. Bioinform..

[24] Taha Said Ahmed S.S., Ahmed Tayeb B. (2017). Isolation and molecular detection of *Listeria monocytogenes* in minced meat, frozen chicken and cheese in Duhok Province, Kurdistan region of Iraq. J. Food Microbiol. Saf. Hyg..

[25] El-Demerdash A.S., Raslan M.T. (2019). Molecular characterization of *Listeria monocytogenes* isolated from different animal-origin food items from urban and rural areas. Adv. Anim. Vet. Sci..

[26] Gelbíčová T., Karpíšková R. (2009). Occurrence and characteristics of *Listeria monocytogenes* in ready-to-eat food from retail market in the Czech Republic. Czech. J. Food Sci..

[27] Oliveira T.S., Varjão L.M., da Silva L.N.N., de Castro Lisboa Pereira R., Hofer E., Vallim D.C., de Castro Almeida R.C. (2018). Listeria monocytogenes at chicken slaughter house: Occurrence, genetic relationship among isolates and evaluation of antimicrobial susceptibility. Food Control..

[28] Mena C., Almeida G., Carneiro L., Teixeira P., Hogg T., Gibbs P.A. (2004). Incidence of *Listeria monocytogenes* in different food products commercialized in Portugal. Food Microbiol..

[29] Akrami-Mohajeri F., Derakhshan Z., Ferrante M., Hamidiyan N., Soleymani M., Conti G.O., Tafti R.D. (2018). The prevalence and antimicrobial resistance of *Listeria* spp in raw milk and traditional dairy products delivered in Yazd, Central Iran (2016). Food Chem. Toxicol..

[30] Basha K.A., Kumar N.R., Das V., Reshmi K., Rao B.M., Lalitha K.V., Joseph T.C. (2019). Prevalence, molecular characterization, genetic heterogeneity and antimicrobial resistance of *Listeria monocytogenes* associated with fish and fishery environment in Kerala, India. Lett. Appl. Microbiol..

[31] Girma Y. (2018). Isolation, Identification and antimicrobial susceptibility of *Listeria* species from raw bovine milk in Debre-Birhan Town, Ethiopia. Ethiop. J. Zoonotic Dis. Public Health.

[32] Somer L., Kashi Y. (2003). A PCR method based on 16S rRNA sequence for simultaneous detection of the genus *Listeria* and the species *Listeria monocytogenes* in food products. J. Food Prot..

[33] Harb O., Elbab G., Shawish R., Mousa W., Abdeen E. (2020). Genetic detection of *Listeria monocytogenes* recovered from fillet fish samples. Alexandria J. Vet. Sci..

[34] Abdellrazeq G., Kamar A., ElHoushy S. (2014). Molecular characterization of *Listeria* species isolated from frozen fish. Alexandria J. Vet. Sci..

[35] Kaur S., Singh R., Sran M.K., Gill J.P.S. (2018). Molecular characterization of *Listeria monocytogenes* in white meat samples from Punjab, India. Indian J. Anim. Res..

[36] Soni D.K., Dubey S.K. (2014). Phylogenetic analysis of the *Listeria monocytogenes* based on sequencing of *16S rRNA* and *hlyA* genes. Mol. Biol. Rep..

[37] Soni D.K., Singh M., Singh D.V., Dubey S.K. (2014). Virulence and genotypic characterization of *Listeria monocytogenes* isolated from vegetable and soil samples. BMC Microbiol..

[38] CFSPH (2019). Listeriosis.

