# Supplementary material for: Prevalence, Antibiogram and Genetic Characterization of Listeria monocytogenes from Food Products in Egypt
Source: Foods. 2021 Jun 15;10(6):1381. doi: 10.3390/foods10061381 (PMC8232309; doi:10.3390/foods10061381)
Supplement: Supplementary file 1 [file foods-10-01381-s001.zip › foods-1267488-supplementary.pdf]

Suppl. Table S1. Biochemical identification of *Listeria monocytogenes* and other *Listeria* species.

		L. monocytogenes	***L. innocua***	***L. ivanovii***	***L. seeligeri***	***L. welshimeri***	***L. grayi***
Carbohydrates fermentation	Dextrose	+	+	+	+	+	+
	Mannitol	−	−	−	−	−	+
	Rhamnose	+	+/−	−	−	+/−	+/−
	Xylose	−	−	+	+	+	−
B-hemolysis		+	−	+	+	−	−
Catalase		+	+	+	+	+	+
Oxidase		−	−	−	−	−	−
CAMP test		+	−	−	−	−	−

Supplementary Table S2. Antimicrobial resistance profiles of *Listeria monocytogenes* isolates from food products.

Sample ID	Source	β-lactams						Fluoroquinolones									
		Amoxicillin-Clavulanic acid (AMC)		Cefotaxime (CTX)		Amoxicillin (AX)		Norfloxacin (NOR)		Ciprofloxacin (CIP)		Levofloxacin (LEV)		Danofloxacin (DA)		Nalidixic acid (NA)	
		Inhibitory zone (mm)	Interpretation	Inhibitory zone (mm)	Interpretation	Inhibitory zone (mm)	Interpretation	Inhibitory zone (mm)	Interpretation	Inhibitory zone (mm)	Interpretation	Inhibitory zone (mm)	Interpretation	Inhibitory zone (mm)	Interpretation	Inhibitory zone (mm)	Interpretation
1	Milk	2.5	0	0.2	0	0.5	0	1.7	1	0.2	0	0.2	0	1.2	(+ , -)	1.1	(+ , -)
2	Milk	2.3	1	0.4	0	1.9	1	1.7	1	2	1	2.5	1	0.7	0	1.4	(+ , -)
3	Milk	2.5	1	2.2	1	2.1	1	.5	0	2	1	1.4	(+ , -)	0.6	0	1.4	(+ , -)
4	Fish fillet	2.3	1	1.9	1	1.9	1	1.9	1	2	1	0.5	0	2.3	1	2.2	1
5	Fish fillet	2.5	0	1.9	1	1.9	1	2	1	1.5	(+ , -)	2.5	1	0	0	0.3	0
6	Fish fillet	2.3	1	0	0	2.3	1	1.2	(+ , -)	0.8	0	2	1	2.4	1	1.2	(+ , -)
7	Fish fillet	0	1	1.4	(+ , -)	0.2	0	2.4	1	2	1	0.2	0	0	0	2.4	1
8	Minced meat	0.4	(+ , -)	2.2	1	0	0	2.4	1	1.4	(+ , -)	2.2	1	0	0	1.2	(+ , -)
9	Minced meat	2.4	0	2.2	1	2	1	2.3	1	0.2	0	2.4	1	2.3	1	1.4	(+ , -)
10	Minced meat	0	1	2.1	1	1.5	(+ , -)	2.6	1	1.8	1	0.2	0	0.5	0	0	0
11	Minced meat	2.4	1	2	1	2.5	1	2.5	1	2.2	1	2.2	1	0.3	0	2.4	1
12	Minced meat	2.5	1	1.1	(+ , -)	1.9	1	2.5	1	2	1	0.3	0	2.5	1	1.2	(+ , -)
13	Minced meat	2.5	1	1.7	1	0	0	2.1	1	2.3	1	2.5	1	2.1	1	0.5	0
14	Minced meat	2.4	1	1.8	1	2.3	1	2.3	1	2.2	1	2.4	1	2.1	1	2.3	1
15	Sausage	2.4	1	1.9	1	2.2	1	2.3	1	0	0	0	0	1.6	(+ , -)	1.5	(+ , -)
16	Sausage	2.5	1	1.8	1	0.3	0	0.3	0	0.3	0	1.2	(+ , -)	2.6	1	1.5	(+ , -)
17	Sausage	2.6	1	2.1	1	2	1	2.3	1	2.2	1	0.3	0	2.4	1	1.3	(+ , -)
Control	*L. monocytogenes* LMEGY1	2.9	1	2.5	1	2.6	1	2.4	1	2.4	1	2.4	1	2.2	1	2	1

Note: 1 = Sensitive; 0 = Resistant; (+, -) = Intermediate

Continued

Sample ID	Source	Aminoglycosides				Macrolides				Tetracycline				Chloramphenicol		Sulfonamides		Glycopeptides	
		Amikacin (AK)		Gentamicin (CN)		Erythromycin (E)		Azithromycin (AZM)		Doxycycline (DO)		Oxytetracycline (T)		Chloramphenicol (C)		Trimethoprim-Sulfamethoxazole (SXT)		Vancomycin (VA)	
		Inhibitory zone (mm)	Interpretation	Inhibitory zone (mm)	Interpretation	Inhibitory zone (mm)	Interpretation	Inhibitory zone (mm)	Interpretation	Inhibitory zone (mm)	Interpretation	Inhibitory zone (mm)	Interpretation	Inhibitory zone (mm)	Interpretation	Inhibitory zone (mm)	Interpretation	Inhibitory zone (mm)	Interpretation
1	Milk	1.9	1	1.8	1	2.2	1	1.3	(+ , -)	0.2	0	0.2	0	1.1	0	0.3	0	2	1
2	Milk	2	1	1.9	1	2.8	1	2	1	0.3	0	0.5	0	1	0	0.3	0	2	1
3	Milk	1.5	(+ , -)	2	1	1.3	(+ , -)	2.2	1	3	1	2.5	1	1.7	(+ , -)	0	0	0.4	0
4	Fish fillet	1.9	1	0.2	0	3	1	0.2	0	3	1	1.4	(+ , -)	2.7	1	3	1	2	1
5	Fish fillet	1	0	1.2	(+ , -)	2.8	1	0.2	0	1.3	(+ , -)	0.2	0	2.5	1	0.5	0	2	1
6	Fish fillet	0.5	0	1.8	1	0.5	0	0	0	0.2	0	0.2	0	0.3	0	3	1	2	1
7	Fish fillet	2.4	1	2	1	2.5	1	1.4	(+ , -)	0.4	0	0.4	0	0.4	0	0.2	0	1.7	(+ , -)
8	Minced meat	2.1	1	0.7	0	0.6	0	1.4	(+ , -)	2.4	1	2.3	1	0.2	0	0.2	0	2.1	1
9	Minced meat	0.8	0	2	1	2.2	1	0.2	0	0	0	0	0	0.2	0	0.2	0	0.2	0
10	Minced meat	2.4	1	0.4	0	2.5	1	0	0	1.6	(+ , -)	2.5	1	0	0	1.4	(+ , -)	2	1
11	Minced meat	1.9	1	2	1	0.5	0	1.4	(+ , -)	0.2	0	0.2	0	0.4	0	0.2	0	2	0
12	Minced meat	1.9	1	1.5	(+ , -)	2.8	1	1.2	(+ , -)	0	0	0.2	0	2.5	1	0	0	2	1
13	Minced meat	2.1	1	2.1	1	3	1	0.7	0	1.4	(+ , -)	0	0	0.2	0	0.7	0	1.4	(+ , -)
14	Minced meat	2	1	2	1	0	0	1.2	(+ , -)	0.5	0	0	0	0	0	0.5	0	2.3	1
15	Sausage	0.3	0	0.2	0	2.6	1	2.4	1	0.5	0	0.6	0	0.2	0	1.4	(+ , -)	0.5	0
16	Sausage	0	0	0	0	0.4	0	0.4	0	0	0	0.7	0	0.6	0	0	0	2.3	1
17	Sausage	2.2	1	2	1	2.8	1	1.3	(+ , -)	0.3	0	0.2	0	1.4	(+ , -)	0.2	0	0.4	0
Control	*L. monocytogenes* LMEGY1	2.8	1	2.5	1	2.9	1	2.7	1	1	0	1.1	0	1.1	0	1.5	0	2.4	1

Note: 1 = Sensitive; 0 = Resistant; (+, -) = Intermediate
